# Pancreatic adenosquamous carcinoma: A population level analysis of epidemiological trends and prognosis

**DOI:** 10.1002/cam4.5700

**Published:** 2023-02-27

**Authors:** Zhihao Huang, Jiakun Wang, Rongguiyi Zhang, Aoxiao He, Shuaiwu Luo, Rongshou Wu, Jianghui Xiong, Min Li, Tao Jin, Enliang Li, Linquan Wu, Wenjun Liao

**Affiliations:** ^1^ Department of General Surgery Second Affiliated Hospital of Nanchang University Nanchang China; ^2^ Office of Science and Technology Administration Second Affiliated Hospital of Nanchang University Nanchang China

**Keywords:** annual percent change, epidemiological trends, pancreatic adenosquamous carcinoma, prognosis, SEER

## Abstract

**Background:**

The incidence and mortality of pancreatic adenosquamous carcinoma (PASC) have received little attention. The goal of our study was to explore the overall epidemiological trend of PASC at the population level.

**Methods:**

The Surveillance, Epidemiology, and End Results database was used to collect the incidence, incidence‐based (IB) mortality, and patient details for PASC from 2000 to 2017. The Joinpoint regression tool was used to examine the trends in incidence and IB mortality. The Kaplan–Meier approach was used for survival analysis. Univariate and multivariate Cox regression analyses were used to determine the independent prognostic factors.

**Results:**

We included 815 patients with PASC in the study. The incidence of PASC continuously increased from 2000 to 2017, with an annual percentage change (APC) of 3.9% (95% CI: 2.2%–5.7%, *p* < 0.05). IB mortality also increased continuously, with an APC of 5.0% (95% CI: 2.5%–7.6%, *p* < 0.05). Multivariate Cox regression analysis revealed that age, treatment, regional lymph node involvement, and tumor size were independent prognostic factors. Nomograms were created for PASC to predict 1‐ and 2‐year survival probabilities, respectively.

**Conclusions:**

The incidence and IB mortality of PASC had a sustained and rapid increase, indicating that the preventive and treatment measures for PASC were not ideal. We must identify the significance of this condition as soon as possible, and commit greater attention and resources to PASC research.

## Graphical abstract

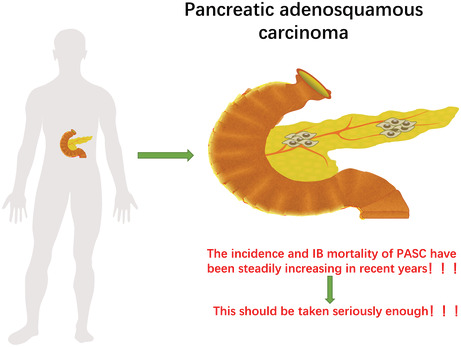

## 1 BACKGROUND

Pancreatic adenosquamous carcinoma (PASC) is a rare subtype of malignant pancreatic exocrine tumor. Other terms include adenoacanthoma, mixed squamous adenocarcinoma, and mucoepidermoid carcinoma, accounting for 1%–4% of pancreatic malignancies.^1^, ^2^, ^3^ The histological characteristics of PASC includes both adenocarcinoma and squamous cells.^4^ Due to the small number of subjects studied, the histological origin of PASC remains contested, with three main theories considered: differentiation theory (two components come from the same cancer group), squamous metaplasia theory, and collision theory (two components merge into a tumor after independent generation).^5^, ^6^, ^7^, ^8^ This situation resulted in inadequate information about the condition, ultimately leading to insufficient attention.

PASC clinically manifests similar to those of pancreatic ductal adenocarcinoma (PDAC), including abdominal pain, jaundice, and weight loss.^9^ Although there are some imaging features suggestive of PASC on enhanced computed tomography (CT) and enhanced magnetic resonance imaging (MRI), the diagnostic sensitivity is low.^10^, ^11^ Currently, the diagnosis of PASC relies on histopathological examination; according to the World Health Organization (WHO) criteria, the proportion of squamous cell carcinoma must be at least 30%.^12^ In addition, there are currently no guidelines for treating patients with PASC. This is done mainly through surgery and confusing chemotherapy regiments.^13^, ^14^, ^15^ Previous studies on PASC were mainly case reports and single‐center retrospective studies with small samples. Most of the studies focused on histopathology. This limited our understanding of the demographic, clinical, and prognostic characteristics of the disease, leading to ineffective management of patients.^6^, ^8^, ^16^, ^17^, ^18^, ^19^, ^20^, ^21^ Due to the dearth of epidemiological studies on PASC, the significance of the disease has been overlooked.

Strengthening the understanding of the epidemiological trends and clinical prognostic characteristics of PASC is essential to standardize its diagnosis and treatment, as well as to evaluate its clinical benefits. Therefore, the purpose of this study was to explore the trend of PASC morbidity and incidence‐based (IB) mortality using the Surveillance, Epidemiology, and End Results database (SEER) to increase attention towards this disease. Additionally, we studied the clinical and prognostic characteristics of PASC.

## 2 MATERIALS AND METHODS

### 2.1 Date source

The SEER database is a reliable source of follow‐up information about cancer patients maintained by the National Cancer Institute (NCI).^22^ Based on the SEER‐18 database, which includes cancer registries representing 28% of the US population, we studied the incidence and mortality of PASC patient from 2000 to 2017, as well as the characteristics of the patients who suffer from this condition.

### 2.2 Study population

**FIGURE 1:**
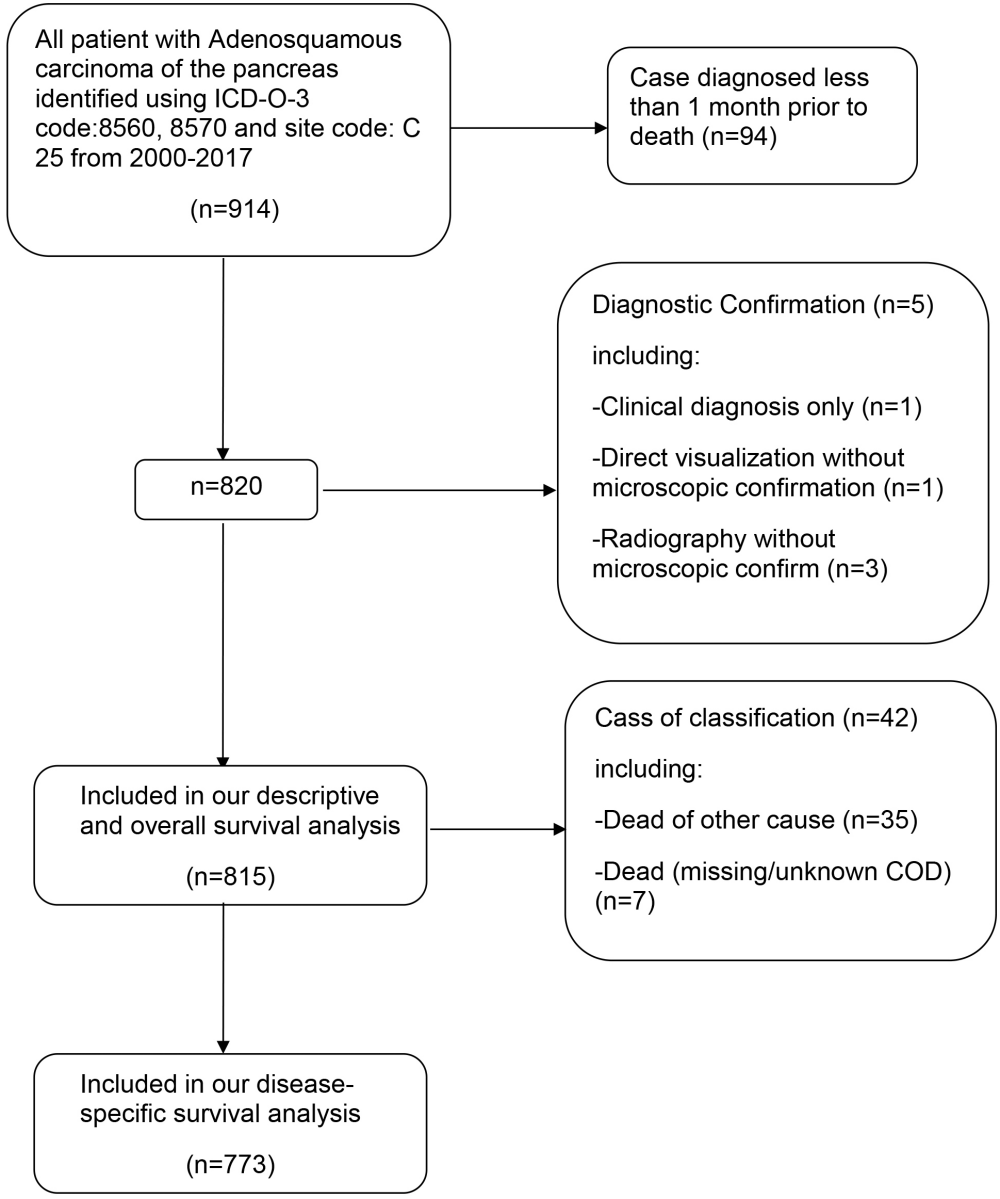
Flow diagram of patient selection out of the total 914 patients in the SEER database 2000–2017.

To identify patients with PASC from 2000 to 2017, we used the International Classification of Diseases for Oncology 3 (ICD‐O‐3) codes (8560,8570) and SEER site codes (C25). In this study, data from patients who died within 1 month of their diagnosis were excluded to avoid cases where the survival time was zero because the smallest unit of survival was the month, rather than the day. In addition, we excluded patients whose diagnosis was not microscopically confirmed by imaging or clinical diagnosis. Overall survival (OS) was analyzed using the case data screened above (Figure 1). Patients who died of non‐pancreatic adenosquamous carcinoma or whose cause of death was unknown were excluded. Disease‐specific survival (DSS) was analyzed using data from the screened patients (Figure 1). We used the same screening method to obtain data from patients with PDAC and pancreatic squamous cell carcinoma (PSCC) for comparison. We included 90,343 patients with PDAC and 306 with PSCC in OS analysis. Additionally, 85,133 patients with PDAC and 286 patients with PSCC were included in DSS analysis. As the American Joint Committee on Cancer Staging Classification has been updated several times during our study period, we used the SEER stage classification, which provides a more consistent classification standard. According to the SEER staging system, tumors are classified as a localized stage that is limited to the primary site, a regional stage that has spread to regional lymph nodes, and a distant stage that has spread to distant tissues and organs.

### 2.3 Statistical analyses

In this study, the SEER*Stat software (version 8.40) was used for collecting data on incidence, IB mortality, and patient characteristics. Since death certificates do not contain histological information about tumors, we studied IB mortality rather than standard mortality.^23^ IB mortality rates were calculated by combining morbidity—and its characteristics at the time of onset—with death certificates. Patients who are categorized as dying from PASC must have been previously diagnosed, not just based on information on the death certificate. In addition, the incidence and IB mortality rates were adjusted for age in the U.S. standard population in 2000.

The joinpoint regression of NCI (version 4.5.01) was used to calculate the annual percent change (APC) and 95% confidence intervals (CIs). The APC method analyzes the trends in incidence and mortality over time, describing the slope, gradient, and direction of each segment. In this method, a logarithmic linear segmentation model is applied, and the incidence and mortality of tumors are assumed to change in a stable percentage compared with the previous year.^24^

We classified the population according to the location of the lesions and used descriptive statistics to summarize the demographic and clinical characteristics of each group. Chi‐square tests were used to compare categorical variables. We used the Kaplan–Meier method to construct the survival curve and log‐rank test to compare survival differences. The Cox proportional hazards model was used to examine factors associated with mortality. Based on the results of Cox regression analysis, nomograms were developed to predict 1‐ and 2‐year survival rate. Moreover, we assessed the prediction discrimination of the nomogram model using the Harrell consistency index (C‐index), which can determine the difference between predicted and actual survival. Calibration curves were calculated to test whether the outcome prediction matched the actual outcome. All *p* values were two‐sided, and values of *p* < 0.05 were considered statistically significant. All statistical analyses were performed using R (version 4.1.3) and GraphPad Prism (version 8.0.2).

## 3 RESULTS

### 3.1 Patient and tumor characteristics

Between 2000 and 2017, 914 patients were diagnosed with PASC. Of these, 815 patients were included in our OS and descriptive analyses, whereas 773 patients were included in our DSS analysis (Figure 1). We divided the study population into four subgroups based on tumor site (head of pancreas, body of pancreas, tail of pancreas, and others) (Table 1). In the study population, the median age at diagnosis was 69 year old (interquartile range, [IQR]: 62–76 year old). There was a similar proportion of males (*n* = 417, 51.2%) and females (*n* = 398, 48.8%) in the overall cohort. Caucasians comprised the vast majority of the patients (*n* = 666, 81.7%). Based on SEER stage, most patients had regional (*n* = 355, 43.6%) and distant PASC (*n* = 375, 46.0%). Localized tumors occurred in a relatively small number of patients (*n* = 68, 8.3%). Based on the degree of differentiation, most patients had poorly differentiated PASC (*n* = 311, 38.2%), followed by moderately differentiated disease (*n* = 115, 14.1%).

**TABLE 1:** Trends in baseline demographic and pathological characteristics of the study population (2004–2017).

Variable	Total	Head of pancreas	Body of pancreas	Tail of pancreas	Other^a^
No. of patients (*n*)	815	368	119	175	153
Median age (years)	69 (62,76)	70 (61.5,76)	69 (60,74)	69 (62,77)	69 (62,75)
Gender, *n* (%)					
Female	398 (51.2)	175 (47.6)	65 (54.6)	86 (49.1)	72 (47.1)
Male	417 (48.8)	193 (52.4)	54 (45.4)	89 (50.9)	81 (52.9)
Race, *n* (%)					
White	666 (81.7)	309 (84.0)	97 (81.5)	135 (77.1)	125 (81.7)
Black	87 (10.7)	35 (9.5)	14 (11.8)	26 (14.9)	12 (7.8)
Other	62 (7.6)	24 (6.5)	8 (6.7)	14 (8)	16 (10.5)
SEER historic stage, *n* (%)					
Localized	68 (8.3)	34 (9.2)	12 (10.1)	13 (7.4)	9 (5.9)
Regional	355 (43.6)	196 (53.3)	47 (39.5)	64 (36.6)	48 (31.4)
Distant	375 (46.0)	130 (35.3)	59 (49.6)	97 (55.4)	89 (58.2)
Unstaged	17 (2.1)	8 (2.2)	1 (0.8)	1 (0.6)	7 (4.6)
Grade, *n* (%)					
Well differentiated	4 (0.5)	1 (0.3)	0 (0)	1 (0.6)	2 (1.3)
Moderately differentiated	115 (14.1)	40 (10.9)	21 (17.6)	35 (20.0)	19 (12.4)
Poorly differentiated	311 (38.2)	152 (41.3)	41 (34.5)	72 (41.1)	46 (30.1)
Undifferentiated	17 (2.1)	11 (3.0)	3 (2.5)	3 (1.7)	0 (0)
Unknown	368 (45.1)	164 (44.5)	54 (45.4)	64 (36.6)	86 (56.2)

^a^ Other group include: Pancreatic duct: 4 individuals, Overlapping lesion of pancreas: 78 individuals, Other specified parts of pancreas: 8 individual, Pancreas: 63 individuals.

As shown in Table 1, the median age of diagnosis in patients with PASC of the head was 70 years old (IQR: 61.5–76 years old). The median age of patients with PASC in the body and tail of the pancreas was same at the time of diagnosis, both 69 years old (IQR, body: 60–74 years old; tail: 62–77 years old). Similar to the general study population, the proportions of males and females with tumors at different sites were approximately equal. Interestingly, the rate of regional disease was significantly higher in patients with PASC of the head than in those with PASC of the body (*p* = 0.009) and tail (*p* < 0.001) of the pancreas. The rate of distant disease was significantly lower in patients with PASC of the head than in those with PASC of the body (*p* = 0.006) and tail (*p* < 0.001) of the pancreas. However, the proportion of moderately differentiated disease was significantly higher in patients with PASC of the head than in patients with PASC of the tail (*p* = 0.004).

Patients who underwent surgery had a higher proportion of regional PASC than those who did not (77.8% vs. 22.5%, *p* < 0.001) (Figure S1A). In contrast, the proportion of distant PASC in patients who received surgical treatment was lower than that in patients without surgical treatment (17.5% vs. 70.0%, *p* < 0.001). In addition, we studied the relationship between tumor size and SEER stage (Figure S1B). Regional PASC was more common in patients with tumors ≤3.5 cm (58.9% vs. 43.9%, *p* = 0.003), and distant PASC was more common in patients with tumors >3.5 cm (49.4% vs. 27.0%, *p* < 0.001).

### 3.2 Overall incidence and IB mortality trends

**FIGURE 2:**
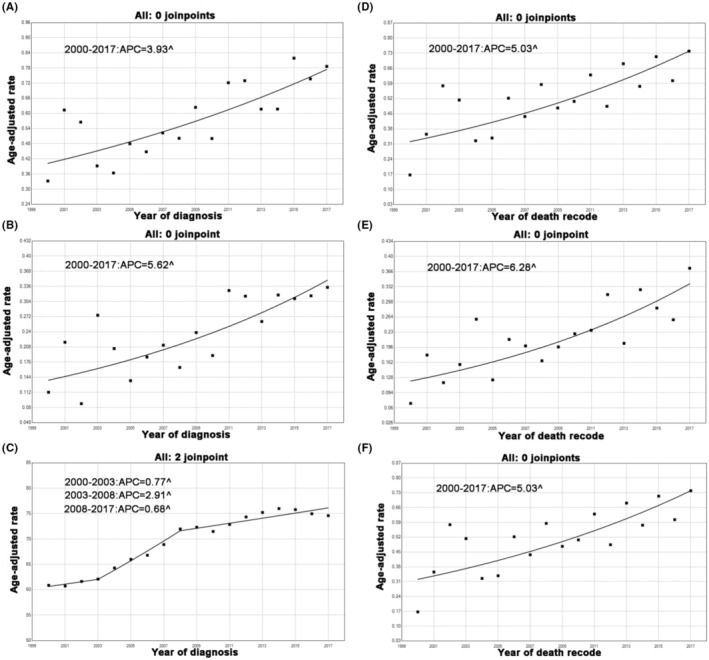
Incidence and IB mortality trends in PASC, PSCC and PDAC overall 2000–2017. (A) Incidence trends in PASC. (B) Incidence trends in PSCC. (C) Incidence trends in PDAC. (D) IB mortality trends in PASC. (E) IB mortality trends in PSCC. (F) IB mortality trends in PDAC. ^ mean that *p* < 0.05.

The incidence of PASC steadily increased during the study period (Figure 2A). The incidence of PASC increased from 0.33 cases per 1,000,000 individuals in 2000 to 0.79 per 1,000,000 individuals in 2017, with an APC of 3.9% (95% CI: 2.2%–5.7%, *p* < 0.05) (i.e., the slope or extent of the increase in incidence). We also analyzed the incidence of PDAC and PSCC for comparison. The incidence of PSCC also showed a consistent upward trend during the study period (Figure 2B). The incidence of PSCC was 0.11 cases per 1,000,000 individuals in 2000, and 0.33 cases per 1,000,000 individuals in 2017. The APC was 5.6% (95% CI: 2.9%–8.4%, *p* < 0.05). Furthermore, the incidence of PDAC increased faster earlier in the study, from 62.14 cases per 1,000,000 individuals in 2000 to 72.00 cases per 1,000,000 individuals in 2002, with an APC of 2.9% (95% CI: 1.6%–4.2%, *p* < 0.05) (Figure. 2C). Since 2008, however, the increase in PDAC rates has been moderate, with an APC of 0.7% (95% CI: 0.3%–1.0%, *p* < 0.05).

During the research period, IB mortality of PASC increased continuously (Figure 2D). The IB mortality rate of PASC increased from 0.17 cases per 1,000,000 individuals in 2000 to 0.74 per 1,000,000 individuals in 2017, with an APC of 5.0% (95% CI: 2.5%–7.6%, *p* < 0.05). Similarly, the IB mortality of PSCC also showed a continuous upward trend, with an APC of 6.3% (95% CI: 3.7%–9.0%, *p* < 0.05) (Figure 2E). The IB mortality of PDAC rapidly increased early in the study period, but the upward trend gradually slowed (Figure 2F). The APC declined from 2.1% (95% CI: 1.2%–2.9%, *p* < 0.05) between 2002 and 2009 to 0.9% (95% CI: 0.3%–1.4%, *p* < 0.05) between 2009 and 2017.

### 3.3 Incidence and IB mortality trends by sex

We split the study population by sex for additional research and found that the incidence of PASC increased steadily in both males and females, with APCs of 3.4% (95% CI: 0.8%–6.1%, *p* < 0.05) and 4.8% (95% CI: 2.6%–7.1%, *p* < 0.05), respectively (Figure S2A). In addition, the incidence was higher in males than in females. The IB mortality of PASC in both males and females also displayed a consistent rising trend over the study period, with APCs of 5.4% (95% CI: 1.3%–9.6%, *p* < 0.05) in males and 5.5% (95% CI: 2.6%–8.5%, *p* < 0.05) in females (Figure S2B).

### 3.4 Incidence and IB mortality trends by stage

We then stratified the study cohort according to the SEER stage. Overall, the incidence of distant and regional PASC was considerably higher than that of localized PASC (Figure S3A). Moreover, the increasing trends in the incidence of PASC were similar at both distant and regional stages, with APCs of 4.1% (95% CI: 1.0%–7.4%, *p* < 0.05) in distant PASC and 4.3% (95% CI: 1.4%–7.3%, *p* < 0.05) in regional PASC. As with incidence, the IB mortality of localized PASC remained the lowest of the three subgroups (Figure S3B). Furthermore, the increasing trends in IB mortality of PASC were also similar at both distant and regional stages, with APCs of 5.0% (95% CI: 1.4%–8.7%, *p* < 0.05) and 5.8% (95% CI: 3.1%–8.7%, *p* < 0.05) in distant and regional PASC, respectively.

### 3.5 Incidence and IB mortality trends by tumor site

Next, we grouped the study cohort according to the tumor site. Overall, the incidence of PASC was highest in the head of the pancreas, followed by the tail of the pancreas, and lowest in the body of the pancreas; both showed a continuous increase over the study period, with APCs of 4.1% (95% CI: 1.9%–6.3%, *p* < 0.05), 5.7% (95% CI: 0.4%–11.3%, *p* < 0.05), and 6.0% (95% CI: 1.7%–10.5%, *p* < 0.05) in the head, tail, and body of the pancreas, respectively (Figure S4A). The IB mortality of PASC at the head of the pancreas was still the highest and showed continuously increasing trend, with an APC of 5.7% (95% CI: 2.4%–9.1%, *p* < 0.05) (Figure S4B). The increasing trend in IB mortality of the pancreatic tail and body was similar, with APCs of 6.1% (95% CI: 1.8%–10.6%, *p* < 0.05) and 5.9% (95% CI: 1.4%–10.6%, *p* < 0.05), respectively.

### 3.6 Long‐term survival outcomes

**FIGURE 3:**
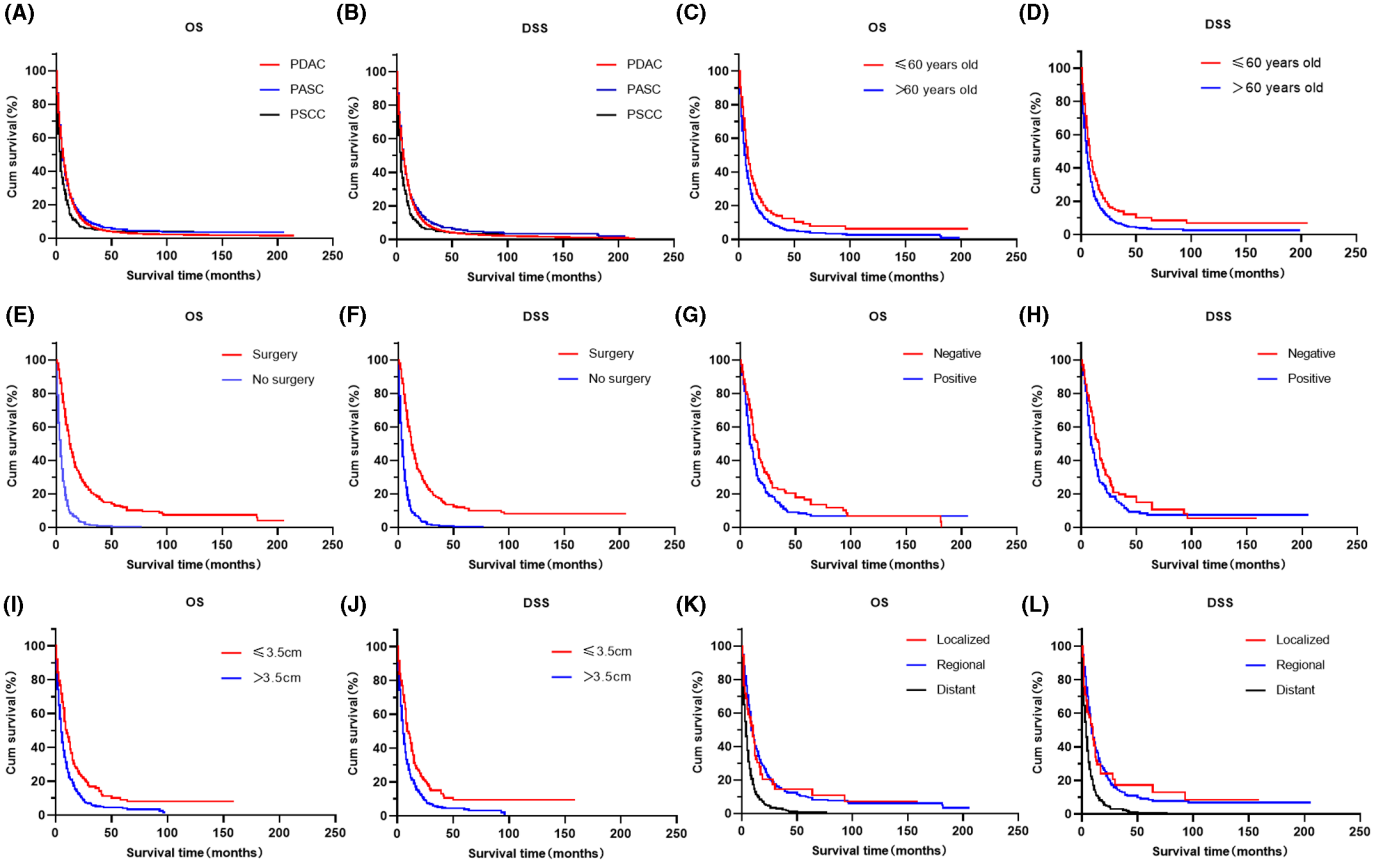
Long‐Term Survival Outcomes using Kaplan–Meier's analysis: (A, B) OS and DSS analysis of PASC, PDAC and PSCC. The OS (*p* < 0.001) and DSS (*p* < 0.001) of patients with PSCC were shorter than those with PASC and PDAC; There were no statistically significant differences in OS (*p* = 0.257) and DSS (*p* = 0.073) among patients with PASC and PDAC. (C, D) OS (*p* < 0.001) and DSS (*p* < 0.001) analysis of PASC stratified by age. (E, F) OS (*p* < 0.001) and DSS (*p* < 0.001) analysis of PASC stratified by treatment. (G, H) OS (*p* < 0.001) and DSS (*p* < 0.001) analysis of PASC stratified by lymph node examination. (I, J) OS (*p* < 0.001) and DSS (*p* < 0.001) analysis of PASC stratified by tumor size. (K, L) OS and DSS analysis of PASC stratified by stage. The OS (*p* < 0.001) and DSS (*p* < 0.001) of patients with distant disease were shorter than those with localized and regional disease; There were no statistically significant differences in OS (*p* = 0.593) and DSS (*p* = 0.815) among patients with localized and regional disease.

The overall median survival of patients with PASC was 6 months (95% CI: 6–7 months), and the disease‐specific median survival of patients with PASC was 6 months (95% CI: 5–7 months). The 1‐ and 2‐year OS were 26.6% and 13.6%, respectively, and the corresponding DSS were 25.8% and 12.7%, respectively. Compared to patients with PASC, there was no statistical difference in the OS and DSS of patients with PDAC, but the OS (median survival 4 vs. 6 months, *p* < 0.001) and DSS (median survival 4 vs. 6 months, *p* < 0.001) of patients with PSCC were significantly worse (Figure 3A,B). Patients older than 60 years had significantly worse OS (*p* < 0.001) and DSS (*p* < 0.001) [Figure 3C,D]. In contrast, patients with PASC who underwent surgery had significantly longer OS (median survival: 12 vs. 4 months, *p* < 0.001) and DSS (median survival: 12 vs. 4 months, *p* < 0.001) than those who did not (Figure 3E,F). Moreover, positive regional lymph nodes and tumors larger than 3.5 cm led to shorter OS (*p* < 0.001) and DSS (*p* < 0.001) (Figure 3G–J). According to SEER stage, the OS (median survival: 10 vs. 5 months, *p* < 0.001) and DSS (median survival 10 vs. 4 months, *p* < 0.001) of patients with localized PASC were better than those of patients with distant PASC (Figure 3K,L). However, there was no difference in OS and DSS between regional and localized PASC patients. There was also no significant difference in OS and DSS among patients with different sexes and different grades of PASC. (Figure S5A–D). Interestingly, OS (median survival 6 vs. 5 months, *p* = 0.017) and DSS (median survival 6 vs. 5 months, *p* = 0.031) were longer in White patients than in Black patients (Figure S5E,F). Furthermore, the location of the tumor along the pancreas did not affect OS and DSS (Figure S5G,H).

We first used COX regression to conduct univariate analysis and found that age at diagnosis, SEER stage, treatment, regional lymph node involvement, and tumor size were significantly correlated with OS (*p* < 0. 1) and DSS (*p* < 0. 1) (Table S1). These factors were then incorporated into the multivariate COX regression analysis (Table 2). Here, age at diagnosis, treatment, regional lymph node involvement, and tumor size were identified as independent prognosticators of OS and DSS.

**TABLE 2:** Multivariate Cox's proportional hazards model assessing factors associated with mortality after diagnosis of pancreatic adenosquamous carcinoma.

	OS	95% CI			DSS	95% CI		
Risk factor	HR^a^	Lower	Upper	*p* Value	HR^a^	Lower	Upper	*p* Value
Age at diagnose (years)								
≦60	Referent				Referent			
>60	1.32	1.06	1.86	0.033	1.35	1.03	1.92	0.039
SEER stage								
Distant	Referent				Referent			
Localized	0.66	0.35	1.20	0.201	0.68	0.35	1.35	0.25
Regional	0.64	0.46	0.90	0.010	0.65	0.46	0.92	0.016
Treatment								
Surgery	Referent				Referent			
No surgery	4.01	2.33	6.90	<0.001	3.88	6.77	3.69	<0.001
Regional lymph nodes								
Negative	Referent				Referent			
Positive	1.51	1.13	2.00	0.006	1.53	1.13	2.08	0.020
Tumor size								
≦3.5 cm	Referent				Referent			
>3.5 cm	1.43	1.07	1.90	0.016	1.41	1.04	1.90	0.027

^a^ HRs >1.0 indicate a higher risk of death.

### 3.7 Construction of the Nomogram

**FIGURE 4:**
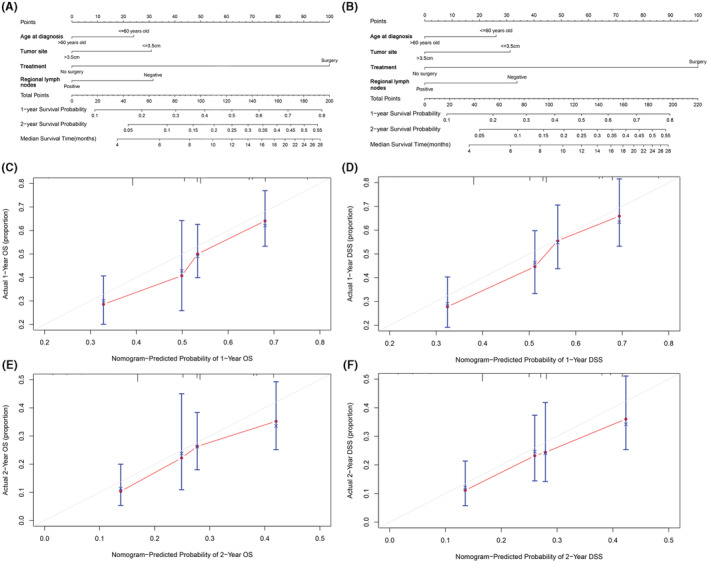
Nomograms to predict survival for patients with PASC and calibration curves of the nomogram. (A) Nomogram to predict 1‐and 2‐year OS for patients with PASC. (B) Nomogram to predict 1‐and 2‐year DSS for patients with PASC. (C) Calibration curves of the nomogram for the prediction of 1‐year OS. (D) Calibration curves of the nomogram for the prediction of 1‐year DSS. (E) Calibration curves of the nomogram for the prediction of 2‐year OS. (F) Calibration curves of the nomogram for the prediction of 2‐year DSS.

The prediction models for OS and DSS contained all of the independent prognostic factors, which were visually represented as nomograms (Figure 4A, B). By adding the related scores for each parameter and projecting the overall score to the bottom level, the probability of 1‐and 2‐year OS and DSS may be calculated. The C‐index values of OS and DSS were 0.724 (95% CI: 0.685–0.763) and 0.695 (95% CI: 0.636–0.753), reflecting the good prediction discrimination of the nomogram models. The calibration curves of internal validation revealed good agreement between predicted and actual OS and DSS at 1, and 2 years (Figure 4C, D, E, F).

## 4 DISCUSSION

Due to the rarity of PASC, studies on its demographic, clinical, and prognostic characteristics of PASC have not been are limited. Kaiser et al. examined the clinical manifestations and prognosis of PASC, while Smoot et al. conducted a retrospective study on PASC resection and palliative treatment. However, the samples were all from a single medical research institution with small sample sizes, involving 91 and 23 patients with PASC, respectively.^17^, ^20^ Hester et al. analyzed the prognosis of PASC using data from multiple research centers. Hue et al. also studied the treatment strategy of PASC through a large cancer database. However, the incidence and mortality of PASC are still unclear, including the changing trend of incidence and mortality.^25^, ^26^ Therefore, a thorough grasp of PASC epidemiological patterns is critical to assist clinicians in making more effective clinical decisions.

In our study, the incidence of PASC increased steadily at a rate of approximately 3.93% per year over the study period, although PASC was relatively rare. This rate of increase is of great concern because it is even faster than that of PDAC. Here, the continuous rise in PASC incidence might imply that prevention has not improved in recent years. Another reason may be that advances in histopathology have led to an increase in the number of diagnoses. Compared with PASC, the overall incidence of PDAC also showed an increasing trend, but the rate of increase has gradually slowed down in recent years. This difference in incidence trends may suggest that precautionary measures for PDAC are more sophisticated and useful than those for PASC, ameliorating the continued rise in incidence. The large number of patients with PDAC has raised concern, leading to the development of preventive measures. The incidence of PASC is increasing much faster than expected; therefore, it should be given sufficient attention. The overall incidence rate of male is significantly higher than that of female, which may suggest that more emphasis should be placed on men when formulating prevention and surveillance strategies.

During the study period, IB mortality rate of PASC also showed a continuous upward trend, with an APC of 5.0%. This might imply that PASC therapy and intervention programs have also not improved significantly in recent years. Interestingly, the overall incidence of PDAC is rising, but the increasing trend has slowed down in recent years. This difference in IB mortality trends may indicate that the treatment methods and strategies for PDAC have made progress and improvement in recent years, as opposed to PASC. As a result, greater resources should be allocated to PASC as well as attempts to establish standardized and focused treatment procedures for these patients. Epidemiological findings could be used to guide both primary prevention through lifestyle modification and secondary prevention through the identification and targeted screening of high‐risk groups with the goal of cancer prevention and early detection. It is important to stress that identification of populations at risk, linkage‐to‐care, improvement in diagnostic tests and implementation of rigorous screening and surveillance strategies may facilitate early‐stage diagnosis of PASC and improve response to currently available treatment options.

Depending on the number of patients with PASC included in the research, previous studies revealed varying survival outcomes, with the overall median survival ranging from 4 to 6 months.^21^, ^26^, ^27^ Consistent with the results of Hester et al. our study found that PASC patients had an overall median survival of 6 months.^26^ Moreover, we found no significant differences in OS and DSS between patients with PASC and PDAC. However, patients with PSCC have shorter OS and DSS than those with PASC and PDAC, despite the poor prognosis for all three diseases.

We discovered that localized and regional PASC significantly delayed OS and DSS compared with distant disease, which is consistent with earlier studies.^27^ However, in our study cohort, patients with distant PASC accounted for the majority. Furthermore, the incidence of distant diseases was significantly higher than that of local and localized diseases, indicating that early diagnosis of PASC was not optimistic. Moreover, according to our results, patients with PASC who underwent surgery had considerably longer OS and DSS than those who did not. However, many patients have an advanced stage when the disease is diagnosed and have lost the opportunity for surgical treatment due to complications or distant metastases.^10^, ^14^, ^27^ The proportion of distal PASC in patients who underwent surgical therapy and survived was significantly lower than that in patients who did not receive surgical treatment in our study (17.5% vs. 70.0%, *p* < 0.001). Therefore, a breakthrough in the early diagnosis of PASC is the main direction of current efforts so that more patients can survive. Exploring comprehensive treatments for patients with advanced disease is also indispensable. At present, there is no exceptional clinical treatment for PASC. Local control and aggressive resection are the first choice treatments for patients with PASC.^28^ In addition, chemotherapy and radiotherapy, as well as combination therapy, are increasingly being used for PASC. In clinical practice, adjuvant chemoradiotherapy is more common than adjuvant radiotherapy or adjuvant chemotherapy. Adjuvant chemoradiotherapy significantly improved the overall survival time of PASC patients compared with adjuvant chemotherapy or adjuvant radiotherapy.^29^ It is important to note that PD‐L1 immune checkpoint is only expressed in squamous carcinoma, and immune checkpoint therapy may be effective in patients with PASC with this characteristic.^14^, ^30^ However, Lee et al. reported that there was no significant difference in the survival of PASC patients between pd‐l1 positive group and pd‐l1 negative group, which may be related to the insensitivity of pancreatic cancer to immunotherapy alone.^14^ Therefore, surgery is the preferred treatment for PASC patients, but adjuvant chemoradiotherapy and comprehensive targeted immunotherapy should also be provided.

According to our analysis, patients with a tumor size >3.5 cm had a worse prognosis. A previous study has also shown that patients with tumors larger than 4 cm tend to have a shorter survival.^26^ We discovered that patients with a tumor size >3.5 cm had a higher frequency of distant PASC than those with a tumor size ≤3.5 cm. In contrast, the proportion of localized disease was smaller in patients with tumors size >3.5 cm than in patients with tumors size ≤3.5 cm. These data imply that tumor size may have an impact on the biological activity of PASCs.

The nomogram has long been employed as an important prediction model for estimating individual survival.^31^ According to our multivariate COX regression analysis, age at diagnosis, SEER stage, treatment, regional lymph node and tumor size were independent prognostic factors affecting OS and DSS of PASC patients. Based on these factors, we developed nomogram 1‐year and 2‐year OS and DSS prediction techniques. The higher C‐index values reflected the good prediction discrimination of the nomogram models. In addition, the calibration curve between the anticipated and actual survival rates for the whole cohort is quite consistent. We may use this model to forecast the transition's survival probability and utilize it as a guide when making clinical decisions.

This is the first research to use a large multi‐center database to study the epidemiological trends of PASC. Despite a detailed analysis of trends in PASC incidence and IB mortality, and subgroup trends by age, grade, and tumor site, there are still some shortcomings in this study. A common problem with large multi‐center databases is selection bias and missing data entry.^32^ In addition, the SEER database does not provide information on serological tests and complications for a more comprehensive analysis.

## 5 CONCLUSIONS

The incidence and IB mortality of PASC have been steadily increasing in recent years, manifesting that the preventive and treatment measures of PASC are not ideal. Therefore, we must identify the significance of this condition as soon as possible and commit greater attention and resources to it. This can help us further understand PASC and develop more targeted and standardized preventive and therapeutic measures. In addition, a nomogram model was developed to forecast the probability of survival of an outcome, which could guide clinical decision making. Further studies on the molecular characteristics of PASC are needed to better determine the biological characteristics and prognosis of the tumor.

## AUTHOR CONTRIBUTIONS

**Zhihao Huang:** Conceptualization (equal); data curation (equal); software (equal); writing – original draft (equal). **Jiakun Wang:** Conceptualization (equal); data curation (equal); formal analysis (equal); software (equal); writing – original draft (equal); writing – review and editing (equal). **Zhang Rongguiyi:** Conceptualization (equal); data curation (equal); formal analysis (equal); writing – original draft (equal). **Aoxiao He:** Methodology (equal); software (equal). **Shuaiwu Luo:** Formal analysis (equal); software (equal). **Rongshou Wu:** Formal analysis (equal); software (equal). **Jianghui Xiong:** Formal analysis (equal); software (equal). **Min Li:** Formal analysis (equal); software (equal). **Tao Jin:** Formal analysis (equal); software (equal). **Enliang Li:** Formal analysis (equal); software (equal). **Linquan Wu:** Conceptualization (equal); writing – review and editing (equal). **Wenjun Liao:** Conceptualization (equal); writing – review and editing (equal).

## FUNDING INFORMATION

This research was supported by the National Natural Science Foundation of China (No. 82060447 and No. 81860431), the Key foundation of Jiangxi Provincial Science and Technology Department (No. 20203BBGL73200), Clinical Research Program for The Second Affiliated Hospital of Nanchang University (2021efyB04), Science and Technology Program for Jiangxi Provincial Health Commission (202210655), Jiangxi Provincial Special Fund for Postgraduate Innovation (YC2021‐B053).

## CONFLICT OF INTEREST STATEMENT

The authors declare that they have no competing interests.

## ETHICS APPROVAL AND CONSENT TO PARTICIPATE

Not applicable.

## CONSENT FOR PUBLICATION

Not applicable.

## Supporting information


Figure S1:

Figure S2:

Figure S3:

Figure S4:

Figure S5:

Table S1:

## Data Availability

The datasets used or analyzed in this study are available from the corresponding author on reasonable request. We have been authorized by the SEER database.
