# Supplementary figures and images for: Pancreatic adenosquamous carcinoma: A population level analysis of epidemiological trends and prognosis
Source: Cancer Med. 2023 Feb 27;12(8):9926–36. doi: 10.1002/cam4.5700 (PMC10166980; doi:10.1002/cam4.5700)

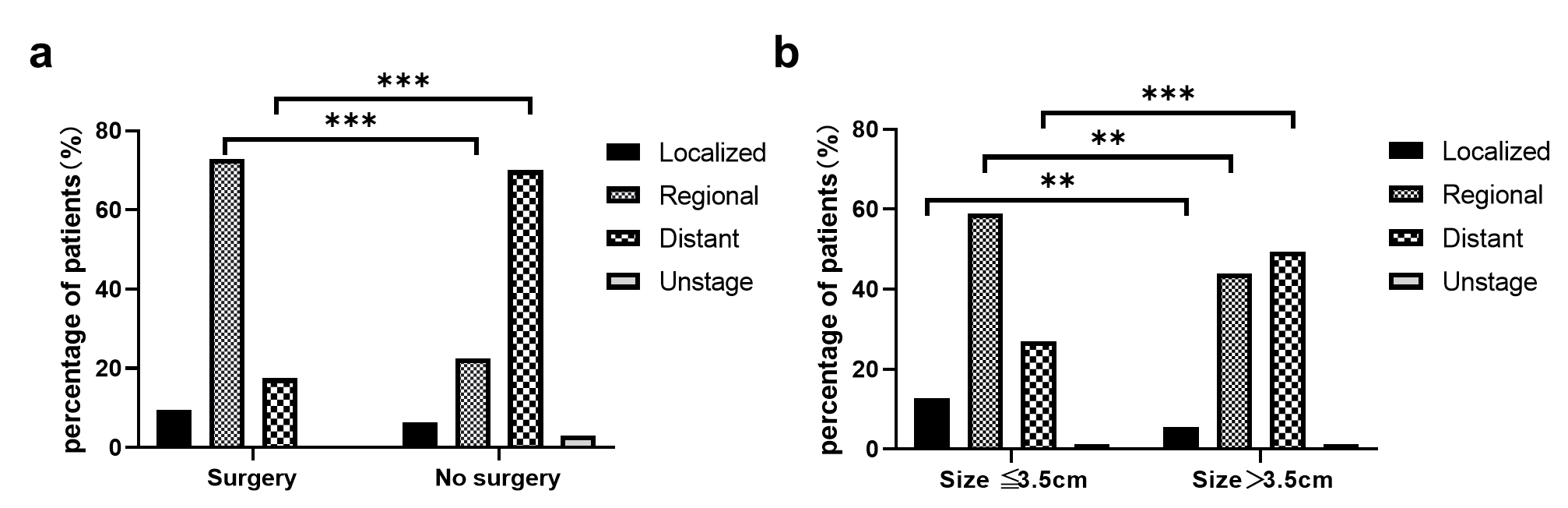

Supplement: Supplementary file 1 — Figure S1: [file CAM4-12-9926-s001.tif]

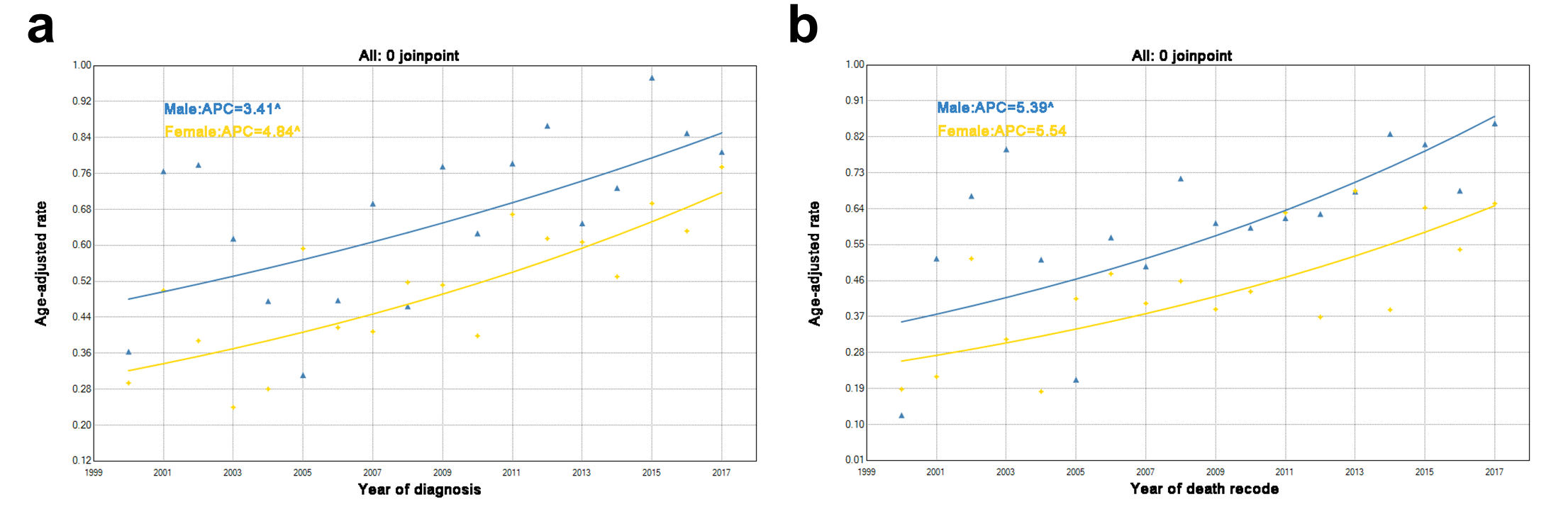

Supplement: Supplementary file 2 — Figure S2: [file CAM4-12-9926-s006.tif]

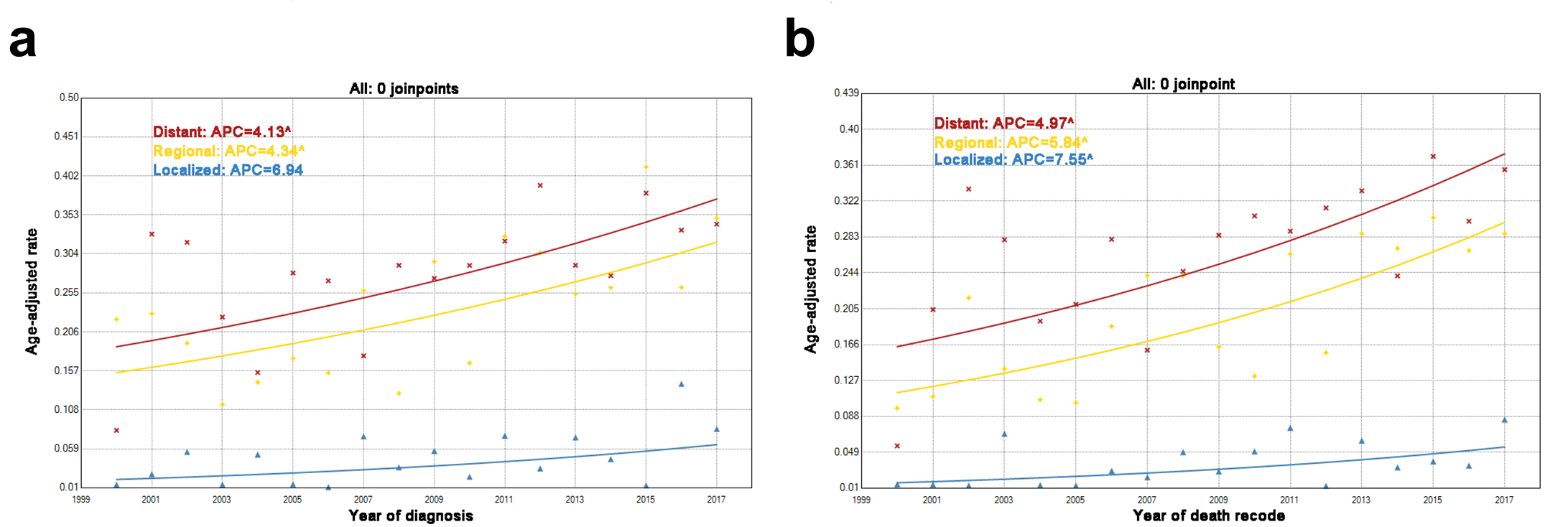

Supplement: Supplementary file 3 — Figure S3: [file CAM4-12-9926-s004.tif]

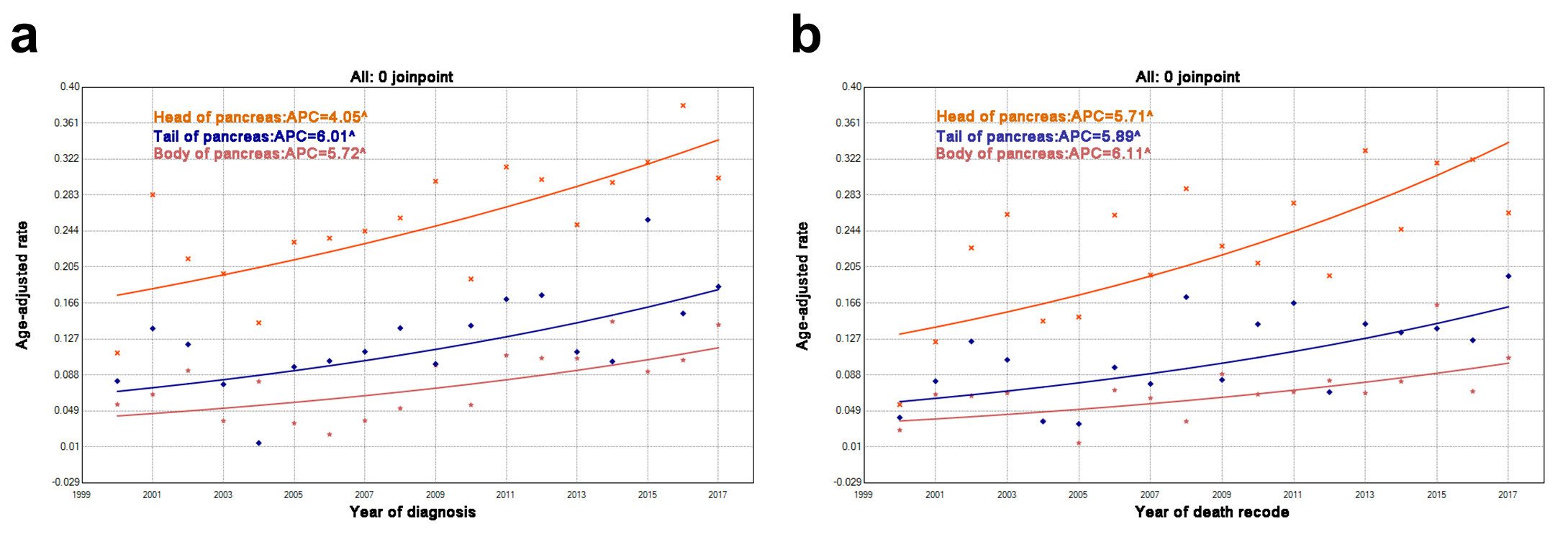

Supplement: Supplementary file 4 — Figure S4: [file CAM4-12-9926-s005.tif]

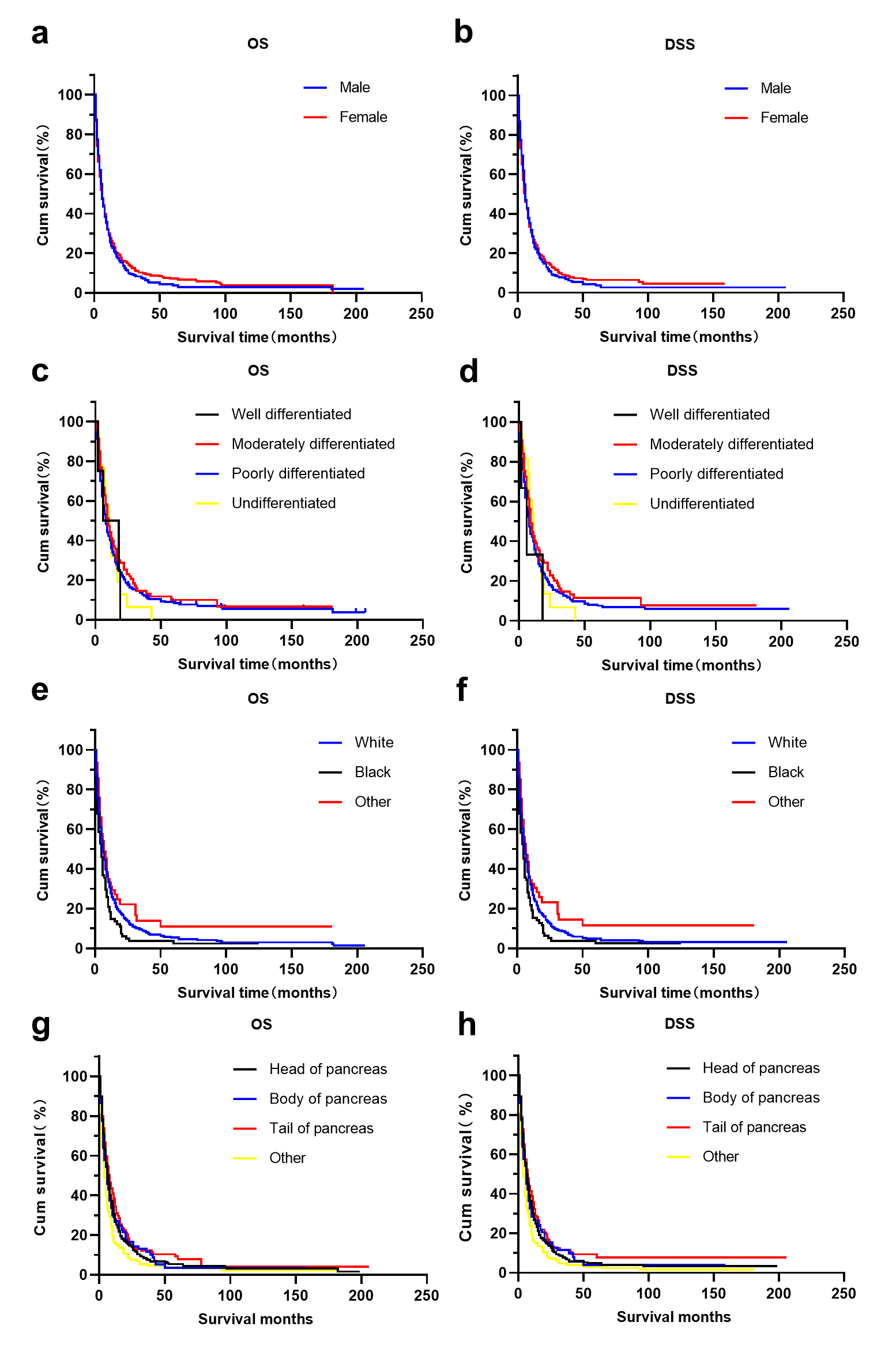

Supplement: Supplementary file 5 — Figure S5: [file CAM4-12-9926-s002.tif]
