# Supplementary material for: Pancreatic adenosquamous carcinoma: A population level analysis of epidemiological trends and prognosis
Source: Cancer Med. 2023 Feb 27;12(8):9926–36. doi: 10.1002/cam4.5700 (PMC10166980; doi:10.1002/cam4.5700)
Supplement: Supplementary file 6 — Table S1: [file CAM4-12-9926-s003.docx]

**TableS1 Univariate Cox’s Proportional Hazards Model Assessing Factors Associated With Mortality After Diagnosis Of Pancreatic Adenosquamous Carcinoma**

|  | **OS** | **95%CI** | |  | **DSS** | **95%CI** | |  |
| --- | --- | --- | --- | --- | --- | --- | --- | --- |
| **Risk Factor** | **HR*** | **Lower** | **Upper** | **P Value** | **HR*** | **Lower** | **Upper** | **P Value** |
| Age at diagnose(years) |  |  |  |  |  |  |  |  |
| ≦60 | Referent |  |  |  | Referent |  |  |  |
| >60 | 1.36 | 1.14 | 1.63 | <0.001 | 1.40 | 1.17 | 1.68 | <0.001 |
| Gender |  |  |  |  |  |  |  |  |
| Male | Referent |  |  |  | Referent |  |  |  |
| Female | 0.95 | 0.82 | 1.11 | 0.530 | 0.98 | 0.84 | 1.14 | 0.745 |
| Race |  |  |  |  |  |  |  |  |
| White | Referent |  |  |  | Referent |  |  |  |
| Black | 1.32 | 1.05 | 1.66 | 0.019 | 1.28 | 1.01 | 1.62 | 0.040 |
| Other | 0.81 | 0.60 | 1.08 | 0.154 | 0.79 | 0.59 | 1.06 | 0.118 |
| SEER stage |  |  |  |  |  |  |  |  |
| Distant | Referent |  |  |  | Referent |  |  |  |
| Localized | 0.48 | 0.36 | 0.65 | <0.001 | 0.48 | 0.35 | 0.65 | <0.001 |
| Regional | 0.45 | 0.38 | 0.53 | <0.001 | 0.46 | 0.39 | 0.55 | <0.001 |
| Treatment |  |  |  |  |  |  |  |  |
| Surgery | Referent |  |  |  | Referent |  |  |  |
| No surgery | 3.22 | 2.73 | 3.80 | <0.001 | 3.12 | 2.63 | 3.69 | <0.001 |
| Grade |  |  |  |  |  |  |  |  |
| Well differentiated | Referent |  |  |  | Referent |  |  |  |
| Moderately differentiated | 0.73 | 0.27 | 1.98 | 0.532 | 0.58 | 1.08 | 1.83 | 0.349 |
| Poorly differentiated | 0.86 | 0.32 | 2.30 | 0.757 | 0.68 | 0.22 | 2.13 | 0.512 |
| Undifferentiated | 0.92 | 0.31 | 2.77 | 0.888 | 0.69 | 0.19 | 2.38 | 0.556 |
| Regional lymph nodes |  |  |  |  |  |  |  |  |
| Negative | Referent |  |  |  | Referent |  |  |  |
| Positive | 1.36 | 1.07 | 1.73 | <0.001 | 1.35 | 1.05 | 1.74 | 0.020 |
| Tumor size |  |  |  |  |  |  |  |  |
| ≦3.5cm | Referent |  |  |  | Referent |  |  |  |
| ＞3.5cm | 1.59 | 1.30 | 1.96 | <0.001 | 1.63 | 1.31 | 2.02 | <0.001 |
| Tumor site |  |  |  |  |  |  |  |  |
| Head of pancreas | Referent |  |  |  | Referent |  |  |  |
| Body of pancreas | 1.01 | 0.81 | 1.26 | 0.920 | 1.01 | 0.80 | 1.27 | 0.953 |
| Tail of pancreas | 0.87 | 0.72 | 1.06 | 0.171 | 0.89 | 0.72 | 1.09 | 0.244 |
| Other | 1.34 | 1.10 | 1.64 | 0.003 | 1.35 | 1.10 | 1.65 | 0.004 |

*HRs greater than 1.0 indicate a higher risk of death
